# Assessment of the Potential of CDK2 Inhibitor NU6140 to Influence the Expression of Pluripotency Markers NANOG, OCT4, and SOX2 in 2102Ep and H9 Cells

**DOI:** 10.1155/2014/280638

**Published:** 2014-11-17

**Authors:** Ade Kallas, Martin Pook, Annika Trei, Toivo Maimets

**Affiliations:** Institute of Molecular and Cell Biology, University of Tartu, Riia 23, 51010 Tartu, Estonia

## Abstract

As cyclin-dependent kinases (CDKs) regulate cell cycle progression and RNA transcription, CDKs are attractive targets for creating cancer cell treatments. In this study we investigated the effects of the small molecular agent NU6140 (inhibits CDK2 and cyclin A interaction) on human embryonic stem (hES) cells and embryonal carcinoma-derived (hEC) cells via the expression of transcription factors responsible for pluripotency. A multiparameter flow cytometric method was used to follow changes in the expression of NANOG, OCT4, and SOX2 together in single cells. Both hES and hEC cells responded to NU6140 treatment by induced apoptosis and a decreased expression of NANOG, OCT4, and SOX2 in surviving cells. A higher sensitivity to NU6140 application in hES than hEC cells was detected. NU6140 treatment arrested hES and hEC cells in the G2 phase and inhibited entry into the M phase as evidenced by no significant increase in histone 3 phosphorylation. When embryoid bodies (EBs) formed from NU6104 treated hES cells were compared to EBs from untreated hES cells differences in ectodermal, endodermal, and mesodermal lineages were found. The results of this study highlight the importance of CDK2 activity in maintaining pluripotency of hES and hEC cells and in differentiation of hES cells.

## 1. Introduction

Cyclin-dependent kinases (CDKs) regulate cell cycle progression and RNA transcription in different cell types. CDKs form complexes that influence several upstream and downstream pathways regulating cell cycle, cell proliferation, and apoptosis. Since alterations in cell cycle progression occur in several malignancies, inhibition of CDKs is regarded as a promising target for cancer treatment. Among the CDKs responsible for cell cycle progression CDK2 is an inherently flexible protein [1] with many conformations needed for interactions with various ligands. CDK2 regulates cell cycle progression by forming (a) cyclin E-CDK2 complexes at the boundary of G1 to S transition and (b) cyclin A-CDK2 complexes for orderly S phase progression and G2 to M phase transition. The inhibition of CDK2 has therefore been an attractive, albeit complicated, task. Using structural-drug design several small molecules and peptides have been developed to target ATP binding subsites or other important binding sites needed for active confirmation of CDK2. Creating highly selective CDK2 compounds is a challenge due to the identity of ATP binding subsites within CDK1, CDK2, and CDK3 molecules; CDK2 also possesses 92% and 80% sequence identity in CDK5 and CDK6 molecules, respectively (RCSB Protein Data Bank code: 1b38). In order to affect CDK2 binding to a specific ligand it would be important therefore to optimize interactions between CDK2 inhibitors and CDK2 residues.

Various specific CDK2 inhibitors have been shown to be effective in inducing apoptosis and reducing proliferation of various cancer cells [2]. In normal cells an induced cell cycle arrest has been shown to be reversible [3, 4]. The properties of CDK2 inhibitors to affect cell cycles are however not completely understood. Only a weak G1 arrest has been observed in CDK2−/− MEFs [5, 6] or after siRNA ablation in established tumor cell lines [7]. An arrest of the cell cycle in the G1 phase has however been detected in cells that have been synchronized and released from a nocodazole-induced mitotic block [8]. Additionally the CDK2 inhibitor flavopiridol was more cytotoxic to transformed cells when treated within the S phase [9]. Cells in certain cell cycle phases are thus likely more sensitive to CDK2 inhibition. Some cancer cells however possess resistance to CDK2 inhibition, as shown by a unique upregulation of CDK2 target proteins and preexisting cellular polyploidy in cancer cells [10].

Among CDK2 inhibitors those with purine-based structures (NU6140 and its derivatives) have shown higher specificity to inhibit CDK2 interaction with cyclin A compared to other interactions (CDK1/cyclin B, CDK4/cyclin D, CDK5/p25, and CDK7/cyclin H) [11, 12]. NU6140 induces apoptosis in HeLa cervical carcinoma cells, arrests cells in the G2/M phase, and reduces cell survival both by itself and in combination with paclitaxel [13]. In epithelial cells however NU6140 has no effect on apoptosis [14]. Exactly how NU6140 affects the cell cycle in carcinoma-derived cells and whether the effect is reversible have remained unclear.

Several specific features of human embryonic stem (hES) cells are of special interest in studying the effect of CDK2 inhibition. First, hES cells are characterized by both unlimited proliferative potential and pluripotency, providing them with the capacity to differentiate into all three cell lineages—ectoderm, endoderm, and mesoderm [15–17]. The capacity to differentiate provides an opportunity to investigate whether CDK2 inhibition could alter the differentiation potential of these cells. Second, hES cells possess a unique cell cycle profile with an abbreviated G1 phase and long S phase [18]. Third, a recent study on phosphoproteome of hES cells during differentiation revealed that CDK2 and Cdc2 activities were central in promoting pluripotency and self-renewal [19]. These three features taken together suggest hES cells can be a useful model for investigating the outcome of CDK2 inhibition. The broad-spectrum inhibitor of CDKs roscovitine has been shown in hES cells to inhibit CDK1, -2, and -5, cause G1/S arrest, accumulate hypophosphorylated Rb, result in smaller cell colonies, and downregulate the pluripotency marker OCT4 [20]. Modulating CDK2 expression by using small interfering RNA (siRNA) can result in the arrest of cells within the G1 phase and induce differentiation of cells into extraembryonic lineages [21]. Expression of p21, a natural inhibitor of CDK2, is associated with G1 arrest in hES cells [22]. Previous studies indicate the important role of CDK2 activity in modulating the hES cell cycle. It is important therefore to clarify how the CDK2 inhibitor NU6140 changes cell cycle and pluripotency of hES cells.

Pluripotency of hES cells is generally maintained by a transcriptional network coordinated by the three transcription factors OCT4, NANOG, and SOX2. Transcription factors (TFs) have been shown to function as cellular rheostats; that is, small changes in TF expression result in big changes in self-renewal and pluripotency of hES cells [23]. hES cell pluripotency and self-renewal are maintained and regulated by cell cycle regulators [24]. Expression of transcription factors characteristic to hES cells has been found in several cancers and is often associated with a poor clinical outcome [25]. Embryonal carcinoma-derived cells (hEC, NTERA-2, BG01V, and 2102Ep) have several advantages as a tool to study stem-cell-like signatures in carcinoma cells as they generally show expressions of proteins and mRNAs specific to hES cells and possess low rates of spontaneous differentiation. The effect of CDK2 inhibitors on the proliferation of various cancer cells has been extensively studied, but less attention has been paid to the ability of CDK2 inhibitors to modulate the expression of transcription factors responsible for pluripotency.

In this study we aimed to characterise the effect of the CDK2 inhibitor NU6140 on the cell cycle in carcinoma-derived cells compared to hES cells. We applied multicolor flow cytometric methods to a single cell analysis of the expression of CDK2 and pluripotency markers in different cell cycle phases. As a consequence of our first experiment another important question we wanted to answer was whether the modulation of transcription factors responsible for pluripotency (NANOG, OCT4, and SOX2) was reversible and long lasting. In addition we also estimated the effect of CDK2 inhibition on the differentiation potential of hES cells.

## 2. Materials and Methods

### 2.1. Ethics Statement

This study was conducted using a commercially available human embryonic stem cell line (WA09-H9, National Stem Cell Bank, Madison, WI, USA); no* in vivo* experiments on animals or humans were performed and therefore approval from an ethics committee was unnecessary.

### 2.2. Cell Culture

The human embryonal carcinoma-derived (hEC) cell line 2102Ep (GlobalStem, USA) was maintained in a DMEM medium (containing glucose 4.5 g/L) (Gibco Life Technologies) containing 10% fetal bovine serum (PAA Laboratories, Linz, Austria) and MEM Nonessential-Amino-Acids-Solution (0.1 mM, Invitrogen, USA).

The human ES cell line H9 (WA09, National Stem Cell Bank, Madison, WI, USA) was maintained on Matrigel (BD Biosciences, San Jose, CA, USA) coated plates in a mTeSR1 maintenance medium (STEMCELL Technologies Inc., Vancouver, Canada) according to the manufacturer's specifications. The mTeSR1 maintenance medium was changed daily. After 3-4 days of growth cell colonies were detached mechanically with a micropipette tip and broken up with gentle pipetting and the resulting individual hES cell clumps were plated onto fresh Matrigel coated plates.

### 2.3. Antibodies and Reagents

Anti-NANOG (PE conjugate), anti-OCT4 (Alexa Fluor 647 conjugate and PerCp-Cy5.5 conjugate), and anti-SOX2 (PerCp-Cy5.5 conjugate) antibodies, plus their isotype control antibodies, were purchased from BD Biosciences. Anti-cleaved-caspase 3 and anti-histone 3 antibodies detecting phosphorylated Serine 10 were purchased from Cell Signaling (USA). Anti-CDK2 antibodies were obtained from Cell Signaling and from Santa Cruz Biotechnology (San Diego, CA, USA). Anti-SOX1 (NorthernLights conjugate, NL-493), anti-OTX-2 (NL-557), anti-Brachyury (NL-557), anti-HAND1 (NL-637), anti-GATA4 (NL-493), and anti-SOX17 (NL-637) antibodies were purchased from R&D Systems (Abingdon, Oxon, UK). Nocodazole and NU6140 (Sigma-Aldrich Chemicals, St. Louis, MO, USA) were dissolved and diluted in DMSO (Sigma-Aldrich Chemicals).

### 2.4. Multivariate Permeabilised-Cell Flow Cytometry and Cell Cycle Analysis

Cells with confluence levels of approximately 60–70% (3-4 days after passage for hES cells, 1-2 days for hEC cells) on 6-well plates were treated for further 24 h with 100 ng/mL nocodazole, 10 *μ*M (or various concentrations) NU6140, or 0.1% DMSO as a control (corresponding to the concentration of DMSO in 10 *μ*M NU6140 solution) or grown without treatment at 37°C in 5% CO_2_ humidified atmosphere. A DMSO concentration of 0.1% used in assays is reported to have less influence in hES cell differentiation than higher concentrations of DMSO [26, 27]. After harvesting with 0.05% trypsin-EDTA solution (PAA Laboratories, Linz, Austria) and washing with PBS, single hES cell suspensions were fixed using 1.6% paraformaldehyde (PFA, Sigma-Aldrich) for 10 min at room temperature (RT) as described for the detection of intracellular phosphoproteins [28, 29]. Cells were then washed with permeabilisation buffer (Foxp3 Staining Buffer Set, e-Biosciences). hES cells were blocked using 2% goat serum (PAA Laboratories) in a permeabilisation buffer (10 min at RT) and stained with appropriate antibodies or their isotype controls for 30 min at RT. For cell cycle analysis cells were further stained with DAPI (Cystain DNA, Partec GmbH, Münster, Germany). Flow cytometry data were acquired with FACSAria using FACSDiva software (BD Biosciences). For analysing 2102Ep cells after fixation with 1.6% PFA cells were permeabilized with ice-cold methanol for 20 min at 4°C, washed with PBS containing 5 mM EDTA, blocked with 6% goat serum in a permeabilisation buffer, and then stained with antibodies as previously described for hES cells. For resuspension of 2102Ep cells the PBS buffer containing 5 mM EDTA was used.

When cells were harvested only for cell cycle analysis they were stained with propidium iodide after ethanol fixation.

**Figure 1:**
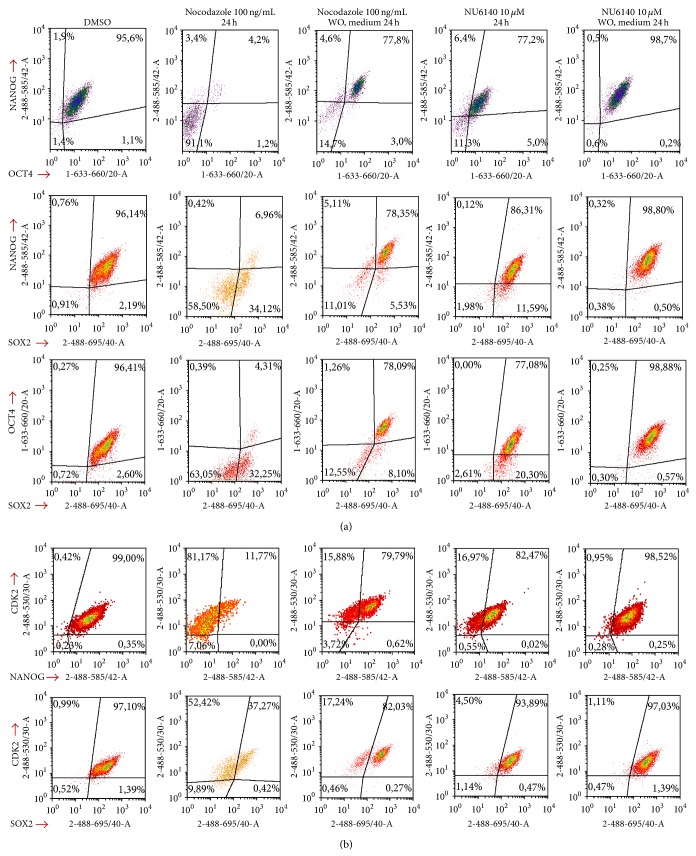
NU6140 treatment compared to nocodazole treatment affects differently the expression of pluripotency markers in hES cells. (a) Flow cytometric analysis of the expression of pluripotency markers NANOG, OCT4, and SOX2 in hES cells treated with nocodazole, NU6140, and DMSO. Fixed and permeabilised cells were stained with anti-NANOG (PE), anti-OCT4 (Alexa Fluor 647), and anti-SOX2 (PerCp Cy5.5 conjugate) antibodies and with DAPI. For analysis cellular debris and doublets were excluded. (b) Correlation between expression of CDK2 with NANOG and SOX2. Fixed and permeabilised cells were stained with anti-CDK2 (Alexa Fluor 488 conjugate), anti-NANOG (PE), and anti-SOX2 (PerCp Cy5.5 conjugate) antibodies and with DAPI.

**Figure 2:**
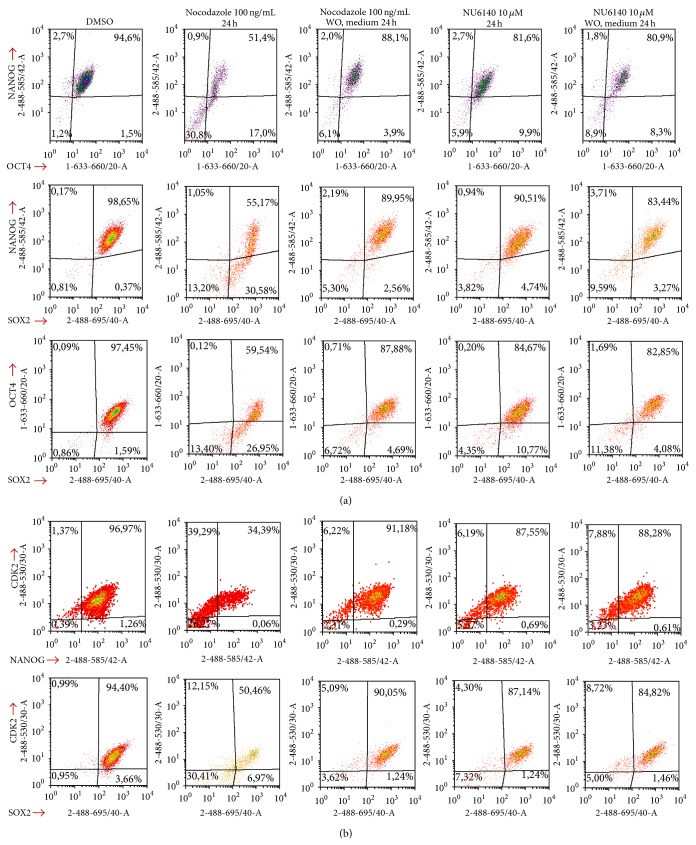
NU6140 treatment compared to nocodazole treatment affects differently the expression of pluripotency markers in 2102Ep carcinoma cells. Expression of pluripotency markers NANOG, OCT4, and SOX2 in nocodazole and NU6140 treated 2102Ep cells as detected by using flow cytometric assays. (a) Fixed and permeabilised (methanol permeabilisation) cells were stained with anti-NANOG (PE), anti-OCT4 (Alexa Fluor 647), and anti-SOX2 (PerCp Cy5.5 conjugate) antibodies and with DAPI. For analysis cellular debris and doublets were excluded. (b) Correlation between expression of CDK2 with NANOG and SOX2. Fixed and permeabilised (methanol permeabilisation) cells were stained with anti-CDK2 (Alexa Fluor 488 conjugate), anti-NANOG (PE), and anti-SOX2 (PerCp Cy5.5 conjugate) antibodies and with DAPI. Results are shown as density plots.

**Figure 3:**
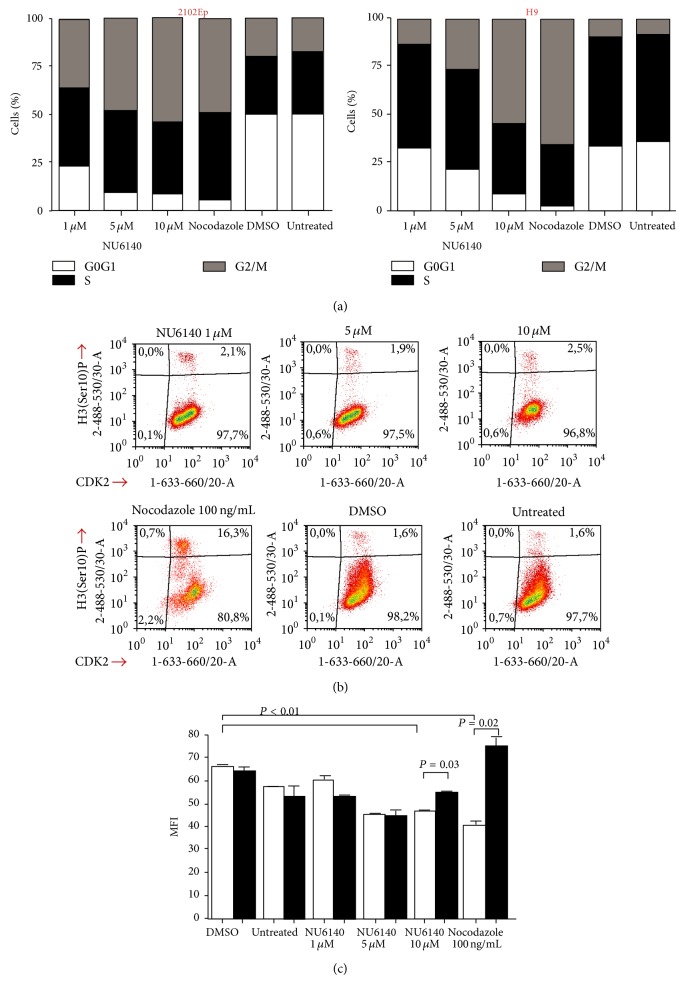
CDK2 inhibitor NU6140 accumulates hES and hEC cells in the G2 phase and nocodazole accumulates cells in the G2/M phase of the cell cycle. Changes in the cell cycle distribution of hES (H9 cells) and hEC (2102Ep cells) cells treated with NU6140, nocodazole, DMSO, or maintenance medium (untreated cells). (a) Distribution of cells within different cell cycle phases. Ethanol fixed and propidium iodide stained cells were analysed by flow cytometry and cell cycle analysis was performed by using ModFit software. (b) Histone 3 phosphorylation at Serine 10 was done using flow cytometric analysis. Results are representative of two independent experiments and shown as density plots. (c) Mean fluorescence intensity (MFI) of CDK2+H3P+ (white empty bar) and CDK2+H3P− (black filled bar) populations as gated in the last density plot (CDK2 expression versus H3 phosphorylation). Results are shown as mean ± SEM (*n* = 4).

**Figure 4:**
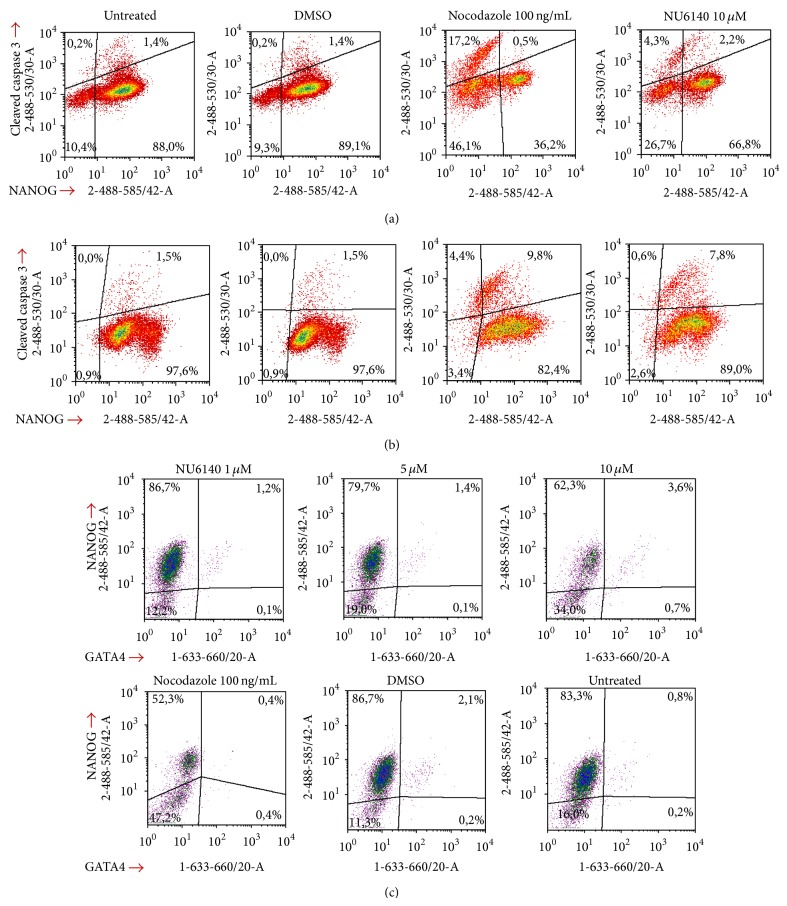
NU6140 treatment increases population of apoptotic hES and hEC cells. Fixed and permeabilised hES and hEC cells were stained with anti-cleaved caspase 3 (Alexa Fluor 488 conjugate) and anti-NANOG (PE). Other hES cells were stained with anti-GATA4 (Alexa Fluor 647) and anti-NANOG (PE conjugate) antibodies and with DAPI. For analysis cellular debris and doublets were excluded. (a, b) The correlation of cleaved-caspase 3 expressing cells and NANOG expression in hES (a) and in hEC (b) cells, respectively. (c) The number of GATA 4 expressing cells and the correlation with NANOG expression in hES cells.

**Figure 5:**
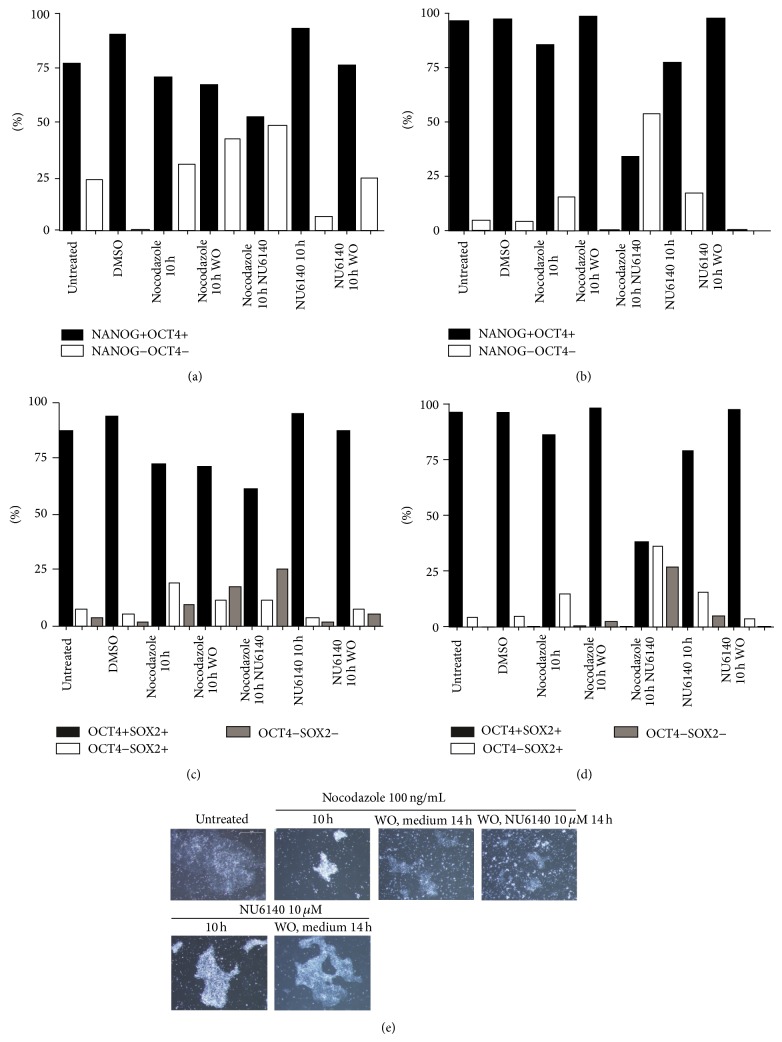
Effect of combined treatment on pluripotency markers expression in hES and hEC cells compared to treatments with nocodazole or NU6140 individually. Nocodazole treated cells (10 h) were washed and further treated with 10 *μ*M NU6140 for 14 h. The coexpression of NANOG/OCT4 and SOX2/OCT4 detected in hEC (a, c) as described in Figure 2 and in hES cells (b, d) as described in Figure 1. (e) Morphological changes in colony structure of hES cells treated with NU6140 or nocodazole.

**Figure 6:**
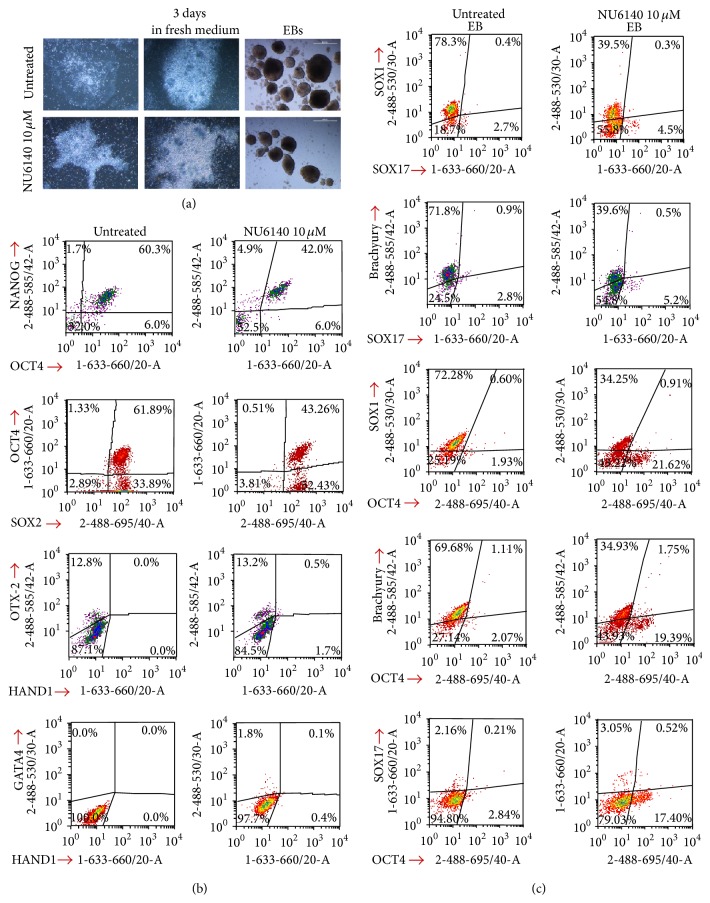
NU6140 treatment causes changes in the colony structure and differentiation potential of hES cells. hES cells were treated with NU6140 (10 *μ*M) for 24 h and then cultured in fresh mTeSR1 maintenance medium for a further 3 days with the medium changed daily. Embryoid bodies were formed from hES cells treated with NU6140 or from untreated hES cells. (a) Morphological changes in colony structure after NU6140 treatment and formation of EBs. (b) Fixed and permeabilised hES cells were stained with anti-OCT4 (Alexa Fluor 647 conjugate), anti-NANOG (PE), and anti-SOX2 (PerCp-Cy5.5) antibodies and with DAPI. Other hES cells were stained with anti-GATA4 (NorthernLights, NL-493 conjugate), anti-OTX-2 (NL-557), anti-HAND1 (NL-637) antibodies and with DAPI. (c) Differentiation into three germ cell layers as detected by flow cytometric assay. Fixed (1.6% PFA) and permeabilised cells from dissociated embryoid bodies (EBs) were stained with anti-SOX1 (NL-493), anti-Brachyury (NL-557), anti-SOX17 (NL-637), and anti-OCT4 (PerCp-Cy5.5) antibodies and with DAPI.

Cell permeabilisation, fixation, staining, and data acquisition for all samples were done on the same day. For more accurate analysis we used sequential selection of cell populations based on size and granularity. Only single cells were selected for detecting cells in different cell cycle phases and only these cells were used to analyse for the expression of various parameters (see Figures 1–6), allowing the minimisation of nonspecific signals.

### 2.5. Western Blot Analysis

Protein samples were electrophoresed on SDS polyacrylamide gel (10%) and transblotted (MiniTransblot Cell, Bio-Rad, Hercules, CA) onto a polyvinylidene difluoride membrane (Millipore, Billerica, MA). Membranes were probed with rabbit anti-NANOG antibodies (Aviva Systems Biology, San Diego, CA, USA), mouse anti-OCT4 antibodies (Santa Cruz Biotechnology), and anti-SOX2 antibodies (Abcam, Cambridge, MA, USA) followed by horseradish peroxidase-conjugated goat anti-rabbit (Cell Signaling) or goat anti-mouse secondary antibodies (LabAs, Tartu, Estonia). A mouse anti-beta-actin antibody (Abcam) was used for detecting loading control. Binding of antibodies was detected with ECL reagent (Western Lightning Plus-ECL, PerkinElmer Inc., Waltham, MA, USA) and exposure of blots by using Bioscpectrum 510 Imaging System with VisionWorks LS software (both from Ultra-Violet Products Ltd., Upland, CA, USA).

### 2.6. Differentiation of hES Cells into Ectoderm, Endoderm, and Mesoderm Lineages

hES cells with confluence levels of approximately 60–70% (3-4 days after passage) on Matrigel were treated with 10 *μ*M NU6140 for 24 h and then a further 72 h with fresh mTeSR1 maintenance medium.

Embryoid bodies (EBs) were formed by growing hES cells in suspension in a low attachment culture plate with Knockout DMEM/F12 media (Gibco, Life Technologies) supplemented by 20% knockout serum replacement, nonessential amino acids (0.1 mM), GlutaMAX (2 mM), and beta-mercaptoethanol (0.1 mM). The ability to form EBs was assessed visually using a microscope with a 37°C heated stage.

### 2.7. Statistical Analysis

A two-tailed paired *t*-test with a confidence interval of 95% was used to analyse the data and was performed with GraphPad Prism 4 software. *P* values less than 0.05 were considered statistically significant. All results are presented as the mean ± standard error. The distribution of cells in each phase of the cell cycle was calculated using ModFit software.

## 3. Results

### 3.1. The Effect of NU6140 on the Expression of Pluripotency Markers Was Different in hEC and hES Cells

First we assessed the ability of the CDK2 inhibitor to influence the expression of pluripotency markers in hES cells. Using flow cytometric analysis the detection of the expression of transcription factors (NANOG, OCT4, and SOX2) was combined with the analysis of cell cycle profiles. In untreated and DMSO treated hES cells the expression of NANOG and OCT4 was high (95.6% and 98.1% NANOG+OCT4+ cells in DMSO treated or untreated cells population). Treatment of cells with nocodazole or NU6140 decreased NANOG+OCT4+ cell population (4.2% and 77% after nocodazole and NU6140 treatment, resp., Figure 1) and increased the number of cells without NANOG and OCT4 expression (91.1% and 11.3% after nocodazole and NU6140 treatment, resp.). The significant correlation between NANOG and OCT4 expression was similar to that detected in our previous study [30]. In DMSO treated cells all NANOG+OCT4+ cells expressed SOX2, indicating that SOX2 is important for pluripotency. In nocodazole treated cells three subpopulations of cells were detected, including those expressing as follows: (i) all three transcription factors; (ii) only SOX2 without NANOG and OCT4 expression (32–34%); (iii) no detectable SOX2 expression (58–63%, Figure 1(a)). NU6140 treatment had a minimal effect on reducing SOX2 expression (2-3% of cells were without SOX2 expression) and the number of surviving cells expressing all three markers (SOX2, NANOG, and OCT4) was higher compared to cells treated with nocodazole (77–86% and 4–7% with NU6140 and nocodazole, resp.). Changes in TFs expression were also detected at the population level using the western blot technique where all adherent cells were collected for analysis (Supplementary Material Figure 1(C) available online at http://dx.doi.org/10.1155/2014/280638). The number of hES cells decreasing after treatment neared significance with NU6140 (*P* = 0.06) and was significant for nocodazole (*P* < 0.01) when they were compared to DMSO treated cells, indicating that both agents were able to inhibit cell proliferation (Supplementary Material Figure 1(A)).

To compare the effects of NU6140 on hES and hEC cells we carried out a similar experiment on 2012Ep cells to the previous experiment on hES cells. For accurate detection of TFs in 2102Ep cells we found that a fixation with 1.6% PFA and permeabilisation with ice-cold methanol resulted in optimal detection of TFs and minimisation of nonspecific signals. A decreased expression of NANOG and OCT4 in surviving cells (51% of NANOG+OCT4+ cells, Figure 2(a)) was detected in nocodazole treated cells compared to those treated with DMSO (94% of NANOG+OCT4+ cells). The expression of NANOG and OCT4 was high in surviving cells after NU6140 treatment (81% compared to 94% NANOG+OCT4+ cells using DMSO, Figure 2(a)). After nocodazole treatment the number of SOX2 expressing cells coexpressing NANOG and OCT4 was lower (55–59% versus 84–90%) and a population of cells expressing only SOX2 higher, when compared with NU6140 treatment (26–30% versus 4–10%). The number of cells without SOX2 expression was higher in nocodazole treated cells (13-14%) than in NU6140 treated cells (3-4%). Western blot analysis of adherent cells showed notable changes in the expression of TFs (Supplementary Material Figure 1(D)). NU6140 and nocodazole were significantly (*P* < 0.05, Supplementary Materials Figure 1(B)) effective in reducing the proliferation of hEC cells. The effects of nocodazole and NU6140 were similar in hES and hEC cells, but hES cells were more sensitive than hEC cells.

Another important issue from a clinical point of view is the recovery of cells after the removal of an inhibitor and the further culture of these cells under normal conditions. We investigated whether the expression of transcription factors is restored after the washout of nocodazole or NU6140 and a further culturing of cells in a fresh medium for 24 h. After release from the nocodazole-block an increase in NANOG and OCT4 expression was detected (from 4% to 77% NANOG+OCT4+ cells, Figure 1(a)) in hES cells, accompanied by a decrease in the subpopulation without NANOG and OCT4 expression (from 91% to 14%, Figure 1(a)). This rather quick recovery in NANOG and OCT4 expressions detected after 24 h was different from our previous report where no significant increase was noticed after removal of nocodazole [30]. Since hES cells in this study were cultured on Matrigel feeder surface but in previous experiments we used hES cells first cultured on MEF feeder and then plated on Matrigel coated plates, these differences in culturing could affect the growing rate, colony formation, and recovery of cells. NU6140 application and its release increased the population of hES cells expressing NANOG and OCT4 from 77% to 98% (Figure 1(a)). Similar changes in SOX2 expression were also detectable in hES cells both with treatment of nocodazole or NU6140 and after their release from these agents. In treatments using nocodazole or NU6140 we detected amongst the released cells a high number of hES cells expressing SOX2 and a smaller subpopulation expressing only SOX2 without NANOG and OCT4 expression. The number of SOX2 expressing cells without NANOG and OCT4 expression changed from 58–63% to 11-12% in nocodazole treated and released cells and from 2-3% to 0.3% in NU6140 treated and released cells.

When 2102Ep (hEC) cells were used instead of hES cells, the removal of nocodazole resulted in similar effects; however removal of NU6140 had a different effect on the expression of all three TFs in hEC cells. The number of cells coexpressing NANOG and OCT4 remained almost the same after NU6140 treatment and removal (81% and 80%, resp., Figure 2(a)), but an increase in the cell subpopulation without SOX2 was noticed after release (from 4% to 11%) though the number of cells expressing all three TFs remained at a similar level throughout.

hES cells were able to restore pluripotency marker expression after the removal of nocodazole and NU6140, with the number of cells remaining unchanged and no significant increase in proliferation of cells detected (Supplementary Material Figure 1). Similar effects of nocodazole treatment and its removal were found in hEC cells, but NU6140 caused long lasting inhibition in the pluripotency marker SOX2 expression. After washout of nocodazole or NU6140, hES cells recovered more efficiently than hEC cells, indicating that different mechanisms regulate the expression of pluripotency markers in hES and hEC cells.

### 3.2. Effect of NU6140 on the Expression of CDK2 Was Different in hEC and hES Cells

As a result of finding the differences in hES and hEC recovery after treatment with NU6140, we investigated the correlation between the levels of pluripotency markers and CDK2 expression in hES and hEC cells. The focus was primarily on the pluripotency markers NANOG and SOX2, because the expression of OCT4 correlated well with the expression of NANOG. First it was confirmed that up to 95% of cells (untreated and control DMSO treated cells) expressed CDK2 and among these two populations of hES cells the following were detected: (i) a majority population expressing NANOG and CDK2 (96–99%) and (ii) a smaller cell population expressing only CDK2 without NANOG expression (1-2%). Nocodazole treatment of hES cells resulted in (i) a decrease in CDK2 and NANOG coexpressing cells (from 99% to 11%); (ii) an increase in cells expressing CDK2 without NANOG expression (from 0.4% to 81%, Figure 1(b)); (iii) an increase in the number of cells without NANOG and CDK2 expression (from 0.2% to 7%, Figure 1(b)). After release from nocodazole arrest and further culturing in fresh medium, all hES cells restored the expression of CDK2, 79% of cells expressed CDK2 and NANOG, and 15% of cells expressed CDK2 without NANOG expression (Figure 1(b)). When NU6140 was used instead of nocodazole, the number of CDK2 and NANOG coexpressing cells increased from 82% to 98% after removal of NU6140 and the CDK2+NANOG− subpopulation decreased from 16% to 0.9%. Cells without CDK2 expression were negative for SOX2 expression and this subpopulation of cells appeared only with nocodazole treatment and not after its removal (Figure 1(b)).

Similar to hES cells almost all untreated 2102Ep cells expressed CDK2. For 2102Ep cells both agents nocodazole and NU6140 downregulated CDK2 expression compared to DMSO treated cells (26–30% and 5–7% CDK2− cells after nocodazole or NU6140 treatment, resp., Figure 2(b)). CDK2 expression was restored after the removal of inhibiting agents nocodazole (2-3% CDK2− cells) or NU6140 (3–5% CDK2− cells). A correlation was found between gradual downregulation of CDK2 expression levels and expression level of NANOG and SOX2 (Figure 2(b)). Nocodazole and NU6140 both affected CDK2 expression in hEC cells, which was different from NU6140 treatment of hES cells where no change in CDK2 expression was detected.

### 3.3. CDK2 Inhibitor NU6140 Accumulates hEC and hES Cells in the G2 Phase

As a consequence of our results showing the effect of NU6140 on CDK2 expression, we analysed the effect of NU6140 on the distribution of cells within the different cell cycle phases in hES and hEC cells, which possess different cell cycle profiles. In hES cells a low concentration (1 *μ*M) of NU6140 had a minimal effect on cell cycles. Higher concentrations (5 and 10 *μ*M) of NU6140 increased the number of hES cells in the G2/M phase and caused a decrease of cells in the G1 phase (Figure 3(a)). Higher concentrations of NU6140 also decreased the number of hES cells within the S phase. After treatment with nocodazole, which arrests cells in the G2/M phases by interfering with the polymerization of microtubules [31], most of the cells were in the G2/M phase (Figure 3(a)).

Similar effects in cell cycle distribution in hES cells were also found for 2102Ep cells with NU6140 and nocodazole treatment (Figure 3(a)). Both NU6140 and nocodazole increased the number of cells within the G2/M phase and decreased it within the G1 phase, with the effect on the S phase minimal.

To distinguish the cells belonging to the G2 or M phases we used the phosphorylation of histone 3 (H3) at Serine 10 as a specific marker of cells in M phase [32]. Nocodazole increased the number of hES cells within the M phase (17% of cells possessed phosphorylated H3 at Serine 10 after nocodazole treatment compared to 2% after DMSO treatment, Figure 3(b)). There was no increase in phosphorylation of H3 at Ser10 (1.9–2.5%) after NU6140 treatment, showing these cells accumulate preferably in the G2, not the M phase; this effect was similar in hEC cells (data not shown).

To find out whether NU6140 or nocodazole had any effect on the expression of CDK2 in cells within the M phase we estimated CDK2 expression level in cells with phosphorylated H3. In hES cells treated with nocodazole the expression level of CDK2 was lower in M phase cells (which expressed both CDK2 and phospho-Ser10-histone) and significantly higher in cells within other cell cycle phases (Figure 3(b)). A similar low level of CDK2 in the M phase was detected by applying increasing concentrations of NU6140. When the mean fluorescence values of the two cell populations (CDK2+ cells with and without phosphorylated H3) were compared, a lower expression of CDK2 in cells within the M phase compared to cells in other cell cycle phases was detected after NU6140 application (Figure 3(c)).

### 3.4. NU6140 Increases Populations of Apoptotic hES and hEC Cells

Since NU6140 treatment decreased the number of adherent hES cells and downregulated expression of pluripotency markers NANOG, OCT4, and SOX2, we asked whether these effects could be due to apoptosis of hES cells. Cleaved caspase 3 was used as a late apoptosis marker to detect apoptotic cells within adherent cell populations expressing the pluripotency marker NANOG. NU6140 treatment induced cleavage of caspase 3 in 4.3% of hES cells without NANOG expression and in 2.2% of cells that still expressed NANOG (Figure 4(a)). Nocodazole induced apoptosis in hES cells (17.7% of cleaved caspase 3 positive cells, Figure 4(a)), but NANOG expression was not detectable in most apoptotic cells.

In hEC cells NU6140 increased the number of apoptotic cells with 0.6% of cleaved caspase 3 positive cells not expressing NANOG and 7.8% of cells still expressing NANOG (Figure 4(b)). In nocodazole treated hEC cells 4.4% cleaved-caspase-3-positive cells were without NANOG expression and 9.8% of cells expressed NANOG.

Since cleaved caspase 3 has been reported to mediate differentiation of hES cells [33], we analyzed NU6140 treated hES cells for the expression of GATA4 as a marker of early differentiation [34, 35]. In our study a small number of GATA4 expressing cells was detected in DMSO treated and untreated hES cells. After NU6140 treatment the number of cells expressing GATA4 slightly increased (4.3% compared to 2.3% GATA4+ cells after NU6140 or DMSO treatment, resp., Figure 4(c)). Most of the GATA4 expressing cells also expressed NANOG in NU6140, DMSO, and untreated cells, indicating that expression of both pluripotency markers and differentiation markers can be detected in the same cell during early stages of differentiation.

### 3.5. NU6140 Was Effective in Reducing NANOG, OCT4, and SOX2 Expression in Nocodazole-Arrested hES and hEC Cells

CDK2 inhibitors have been reported to possess the ability to arrest cells in the G1 phase after synchronization with a nocodazole-induced mitotic block [8]. We tested the effect of NU6140 on nocodazole-arrested cells after a shortened treatment period with nocodazole (10 h instead of 24 h) in order to obtain a higher number of surviving cells. In both hES and hEC cells the number of cells coexpressing NANOG and OCT4 decreased slightly as a result of nocodazole arrest (Figure 5). After careful washing with fresh mTeSR1 maintenance medium cells were treated for a further 14 h with a medium containing 10 *μ*M NU6140. This further treatment of cells with NU6140 decreased more efficiently the expression of NANOG and OCT4. Combined treatment with nocodazole followed by NU6140 resulted in 25% hES and 26% hEC cells without expression of any of three TFs (NANOG−OCT4−SOX2− cells) among surviving cells. Treatments with nocodazole or NU6140 alone were not as effective in decreasing expression of all three TFs. Combined treatment with nocodazole followed by NU6140 was more effective in decreasing expression of all three TFs in hES cells than hEC cells, though the tendencies were similar. We also tested the recovery of cells to express NANOG, OCT4, and SOX2 after washout of nocodazole or NU6140 after 10 h treatments (Figure 5) and results were similar to those obtained with 24 h treatment period (Figures 1(a), and 2(a)).

Figure 5(e) shows the changes in hES cell colony structures after treatment with nocodazole and NU6140. Nocodazole treatment caused significant changes in colony structure and the surviving cells possessed specific characteristics of cells arrested in M phase (round-shape larger cells). After removal of nocodazole fewer round-shape cells were detected and the formation of new small colonies was recognized. NU6140 treatment following nocodazole treatment did not reverse the effect of nocodazole, but rounded cells were detectable and the remaining colonies were similar to those after treatment with NU6140 alone. NU6140 treatment alone removed the single-cell layer from the colony edges and cells located in the center of the multicell layer survived. After removal of NU6140 hES cell colonies consisted only of multicell layers. Cells treated with NU6140 had a compact, tightly packed colony structure that appeared to protect hES cells and support cell survival (Figure 5(e)).

### 3.6. Embryoid Body Formation of hES Cells Occurs after Treatment with NU6140

The next important question to answer was whether NU6140 treated and released hES cells could maintain pluripotency and form colonies. It is known that colony formation consists of 4 stages: attachment, migration, aggregation, and formation [36]. After removal of NU6140 and a further 3 days of culture no further propagation in colony formation was noticed (Figure 6(a)). When hES cells were analysed for expression of pluripotency factors, 42% of cells expressed NANOG, OCT4, and SOX2 3 days after treatment with NU6140. Among untreated hES cells cultured for the same time period 60% of hES cells expressed all three TFs (Figure 6(b)). In order to distinguish undifferentiated cells from differentiated cells, differentiation markers GATA4 (endodermal lineage), OTX-2 (ectodermal lineage), and HAND1 (mesodermal lineage) were used. OTX-2 expressing cells (13.2%) and some GATA4 expressing cells (1.8%) were detected after release from NU6140 and further culture for 3 days, indicating some differentiation process had already started. In comparison, among untreated cells cultured for the same time period 12.8% OTX-2 expressing cells were detected, but no GATA4 and HAND1 expressing cells. In both untreated and NU6140 treated cells differentiation into ectodermal lineage (OTX-2 expression) was detected, when hES cells were cultured in conditions which supported pluripotent state.

Next we applied common embryoid bodies (EBs) formation protocols in order to characterize the differentiation ability of NU6140 treated and released hES cells. NU6140 treatment of hES cells had no effect on formation of EBs (Figure 6(a)). After 4 days, embryoid bodies were analysed for the expression of ectoderm (SOX1), mesoderm (Brachyury), and endoderm (SOX17) markers and pluripotency marker OCT4 simultaneously by using flow cytometric method. We could detect lower number of ectodermal marker SOX1 expressing cells from EBs formed from NU6140 treated cells compared to EBs formed from untreated hES cells (39% versus 78%, Figure 6(c)). The number of mesodermal marker Brachyury expressing cells was also lower in EBs formed from NU6140 treated cells (39% versus 71% of untreated EBs). Endodermal marker SOX17 expression was detected in 4.5% of EBs formed from NU6140 treated hES cells, but in untreated EBs SOX17 expression was detected only in 2.7% of cells. The number of OCT4 expressing cells was higher in EBs formed from NU6140 treated hES cells (17–21%) than from untreated hES cells (1.9–2.8%). OCT4 expression was not detected in these cells, which expressed Brachyury, SOX1, or SOX17 (Figure 6(c)). The distribution of cells within cell cycle phases was similar in both EBs formed from NU6140 treated hES cells or from untreated cells (62%, 23%, and 15% versus 61%, 18%, and 21% in G0G1, S, and G2/M phases, resp.).

## 4. Discussion

Flexibility of CDK2 molecules allows interactions with various ligands to regulate progression and proper dynamics of cell cycles. In this study we focused on the small molecule NU6140, which competes with ATP for binding to the complex of CDK2 and cyclin A [11, 12]. Our experiments using NU6140 treatment focused on changes in the expression patterns of cell pluripotency markers for human embryonic H9 stem cells (hES) and embryonal carcinoma-derived 2102Ep cells (hEC). We found that both hES and hEC cells were sensitive in suppressing the expression of NANOG and OCT4 with nocodazole or NU6140, whereas the effect on SOX2 expression was more pronounced after nocodazole treatment. After the removal of nocodazole or NU6140, the expression of NANOG, OCT4, and SOX2 was restored in hES cells; a similar tendency was detected in hEC cells after nocodazole treatment and release. CDK2 inhibition with NU6140 influenced the ability of hES cells to differentiate. Analysing embryoid bodies (EBs) revealed that differentiation into ectodermal and mesodermal lineages was affected in EBs formed from NU6140 treated hES cells compared to EBs from untreated hES cells.

Several CDK inhibitors have been shown to be effective in inhibiting the proliferation of and inducing apoptosis in various cancer cells [37]. Our finding that application of NU6140 also increased apoptosis and inhibited growth of hES and hEC cells was therefore expected. The effect of NU6140 on the expression of pluripotency markers SOX2, NANOG, and OCT4 had not however been reported before this study. The expression of stem-cell-like TFs has been found in many cancers with poor treatment outcomes [25]. It is therefore important to understand the abilities of potential medical agents in modulating the expression profiles of stem-cell-like TFs using* in vitro* testing. The differences in sensitivity to NU6140 in hES and hEC cells found in this study point to the different roles of CDK2-cyclin A complex formation and its importance in regulating the expression of the three transcription factors.

Treatment with NU6140 had an influence on the differentiation ability of hES cells. Cells of all three germ layers were detected in embryoid bodies (EBs) formed from NU6140 treated hES cells; however commitment into the endodermal lineage was somewhat higher and ectoderm and mesoderm lineages lower when compared to EBs from untreated cells. B**y** applying a flow cytometric method to the analysis of EBs we were able to simultaneously detect both differentiation and pluripotency markers in single cells. Before initiation of EBs and three days after release from NU6140, hES cells displayed a tendency to differentiate with decreasing expression of NANOG and OCT4 (43%) and with increased expression of early ectodermal marker OTX-2 (13%). The increase of OTX-2 expression in EBs has been reported to coincide with the downregulation of the pluripotency marker OCT4 [38]. We found in this study that up to 20% of EBs formed from NU6140 treated cells expressed OCT4 by day 4, whilst only 2-3% EBs from untreated cells expressed OCT4. Cells expressing SOX1 (ectodermal marker), SOX17 (endodermal marker), or Brachyury (mesodermal marker) did not express OCT4, indicating that EBs contained differentiated as well as some OCT4 expressing cells. Another study found a long persistence in the expression of the pluripotency marker OCT4 in differentiating EBs by day 12 after initiation of EBs [39]. Our finding that differentiation and pluripotency markers can be detected during the early stages of cell differentiation accords with our previous study that revealed a gradual decrease in the expression of pluripotency markers which was accompanied by a gradual increase in the expression of endodermal markers [40]. The differences we found in commitment of cells into endodermal, ectodermal, and mesodermal lineages between untreated hES cells and those treated with NU6140 indicates that inhibition of CDK2 with NU6140 may have long term consequences. Treatment of hES cells with NU6140 might also induce epigenic changes, but further studies are required to answer this question.

It has been reported that cells arrested and released from the M phase, as well as cells within the S phase, are more sensitive to CDK2 inhibitors [8, 9]. Results of our study confirm that hES cells have a longer S phase and are more sensitive to treatment with NU6140 than hEC cells. We also tested combined treatments where cells were first treated with nocodazole (arrests cells in M phase) and then with NU6140. A combined treatment of nocodazole and then NU6140 was more efficient in decreasing expression of NANOG, OCT4, and SOX2 in both hES and hEC cells than either treatment alone. hES cells however possessed higher sensitivity than hEC cells to treatments with individual agents or agents combined.

There are several differences between hES and hEC cells that might explain their different sensitivity. Higher expression levels of SOX2 and OCT4 have been found in hEC cells, with expression of OCT4 isoforms (OCT4A, OCT4B, etc.) in hEC cells [41, 42], overexpression of SOX2 in some hEC cell lines [43], and a low differentiation ability indicating that the control of pluripotency factors is more complicated in hEC cells than in hES cells. In hES cells the miRNAs of 371/372/373 and 302 a–d clusters associated with pluripotency are suppressed in cardiac or neural-lineage induction [44]. In testicular germ cell tumors the presence of miRNA-372 and miRNA-373 has been reported, whereas a miRNA302 cluster was undetected [45]. These authors [45] also reported that miRNA-372 and miRNA-373 can neutralize p53-mediated CDK inhibition and allow the growth of tumor cells. Taken together all these differences at various levels of regulating pluripotency indicate that the expression of TFs in hEC cells is not as tightly controlled as it is in hES cells.

In both hES and hEC cells a correlation between the expressions of CDK2 and SOX2 was found in surviving cells after treatment with NU6140 and most of cells expressing SOX2 also expressed CDK2. Our findings argue that CDK2 expression is needed for transcription factor expression and any alterations in activity/expression influence firstly NANOG and OCT4 and then SOX2 expression in hES cells, pointing to possible additional roles of SOX2 in cell self-renewal and proliferation. A gradual downregulation of CDK2 and SOX2 was detected in nocodazole or NU6140 treated hES and hEC cells. Additionally only a few GATA4 expressing cells were detected after NU6140 treatment of hES cells, which could indicate that CDK2 activity is needed for cell survival and proper differentiation. Analysis of hES cell phosphorylation dynamics during differentiation into extraembryonic lineages (endoderm, etc.) has shown that high expression of transcription factors and normal cell cycle profiles are crucial for initiation of hES cell differentiation [19].

In this study we have shown that the expression of CDK2 in M phase arrested cells (nocodazole treatment) was lower than in normally cycling cells. In untreated cells and in DMSO treated cells we detected similar expression levels of CDK2 independently of the cell cycle phases. It is possible that mammalian cells can tolerate loss of CDK2 (by additional support from other CDKs as CDK1, CDK4, or CDK6), but CDK2 activity is required for cell proliferation [46]. CDK1/cyclin A complexes have been specifically implicated in attenuating the expression of histone genes at the end of the S phase [47]. In solid tumors, however, including breast cancers, the overexpression of cyclin E and overexpression of cyclin A have been linked with adverse outcomes [48]. It has been shown that paclitaxel (which disrupts microtubule dynamic and induces mitotic arrest) can increase specific activity in CDK1 cells, though some cells are resistant. Baseline CDK2 activity is also higher in tumors sensitive to paclitaxel [49]. CDK1 is reported to be important for the regulation of mitosis [50]. Both CDK1 and CDK2 could therefore be required for proper M phase entry. In our study M phase arrested cells (application of nocodazole) decreased expression of NANOG, OCT4, and SOX2 in both hES and hEC cells. Release from nocodazole-arrest however restored expression of all three TFs in surviving cells. There was no increase though in the number of surviving cells, indicating that these cells, which had no detectable CDK2 and SOX2 expression, could not survive. Why NU6140 treatment and its removal caused a long lasting effect on decreasing expression of TFs in further colony formation could be explained by the gradual downregulation of CDK2 expression. The effects of NU6140 to inhibit other interactions of various CDKs might also contribute to inhibiting the expression of pluripotency factors and needs further investigation.

The effects of CDK2 inhibitor NU6140 to increase apoptosis and accumulate cells in the G2/M phase has been reported earlier for HeLa cervical carcinoma cells [13]. A novel finding of our study was that histone 3 phosphorylation decreased after treatment with NU6140 in both hES cells and hEC cells, indicating that NU6140 inhibits entry into the M phase and arrests cells in the G2 phase. As NU6140 inhibits the activity of CDK2 by competitive binding to the ATP site [11, 12] and we found NU6140 did not rapidly downregulate CDK2 expression, CDK2 activity but not the expression level is important for cells to proceed from the G2 to M phase of the cell cycle. Another broad-spectrum CDK inhibitor (roscovitine) has also been shown to decrease the expression of mitotic control genes and prevent the entry of HT29 human colon cancer cells into mitosis [51].

Targeting TF expression in cancer cells is a promising and attractive method for finding new treatment options. It has been shown that knockdown of OCT4 and NANOG in pancreatic cancer cells reduced their proliferation, migration, invasion, chemoresistance, and tumorigenesis [52]. According to the results of our study the expression of SOX2 could be the target of choice to eliminate cancer cells with stem-cell-like properties. Recently it has been shown that inhibition of SOX2 reduced the proliferation and migration of gastric cancer cells, increased their apoptosis, induced changes in cell cycle* in vitro*, and reduced their* in vivo* tumorigenic potential [53]. For the effective elimination of cancer cells with stem-cell-like properties a combined treatment of agents may be more effective, as successfully shown with nocodazole and NU6140 in our study. We also produced a screening method to detect expression patterns of three TFs by using multicolor flow cytometric analysis. Multicolor flow cytometric analysis could be a useful tool for screening different potential medical agents and their combinations for the treatment of hES cells and cancer cells* in vitro*. By applying the methods described in this study knowledge critical to create novel approaches to eliminate cancer cells with stem-cell-properties might be obtained.

## 5. Conclusions

The results of this study point to a connection between CDK2-cyclin A complex activity and self-renewal and pluripotency maintenance in hES and in hEC cells. Modulating the activity of this interaction with NU6140 decreased the expression of transcription factors NANOG, OCT4, and SOX2. hES cells were more sensitive than hEC cells to inhibiting agent NU6140. In embryoid bodies formed from NU6140 treated hES cells commitment to ectodermal and mesodermal lineages was affected compared to embryoid bodies formed from untreated cells. A combined treatment (cells arrested with nocodazole and then treated with NU6140) was more efficient in decreasing the expression of all three transcription factors than treatment with nocodazole or NU6140 individually in hES and hEC cells. In addition, applied multiparameter flow cytometric methods for simultaneous detection of pluripotency and differentiation markers in single cells could be a useful tool for* in vitro* screening in order to design novel medical agents to modulate stem-cell-like properties of cancer cells.

## Supplementary Material

Effects of NU6140 and nocodazole on hES and hEC cells survival and the expression of pluripotency markers NANOG, OCT4, SOX2 as detected by Western blotting method.
