# Supplementary figures and images for: Assessment of the Potential of CDK2 Inhibitor NU6140 to Influence the Expression of Pluripotency Markers NANOG, OCT4, and SOX2 in 2102Ep and H9 Cells
Source: Int J Cell Biol. 2014 Nov 17;2014:280638. doi: 10.1155/2014/280638 (PMC4248398; doi:10.1155/2014/280638)

**A**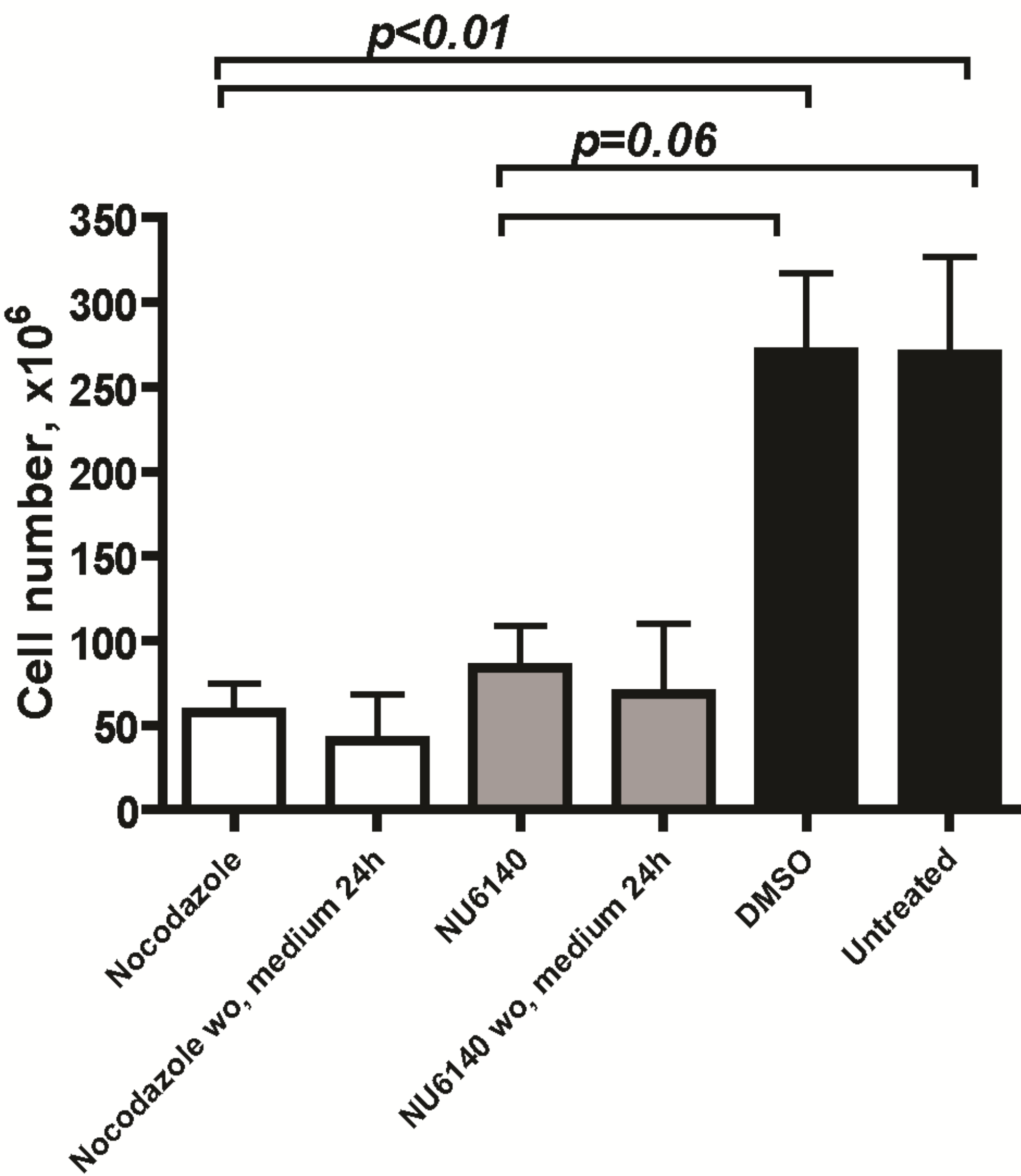**B**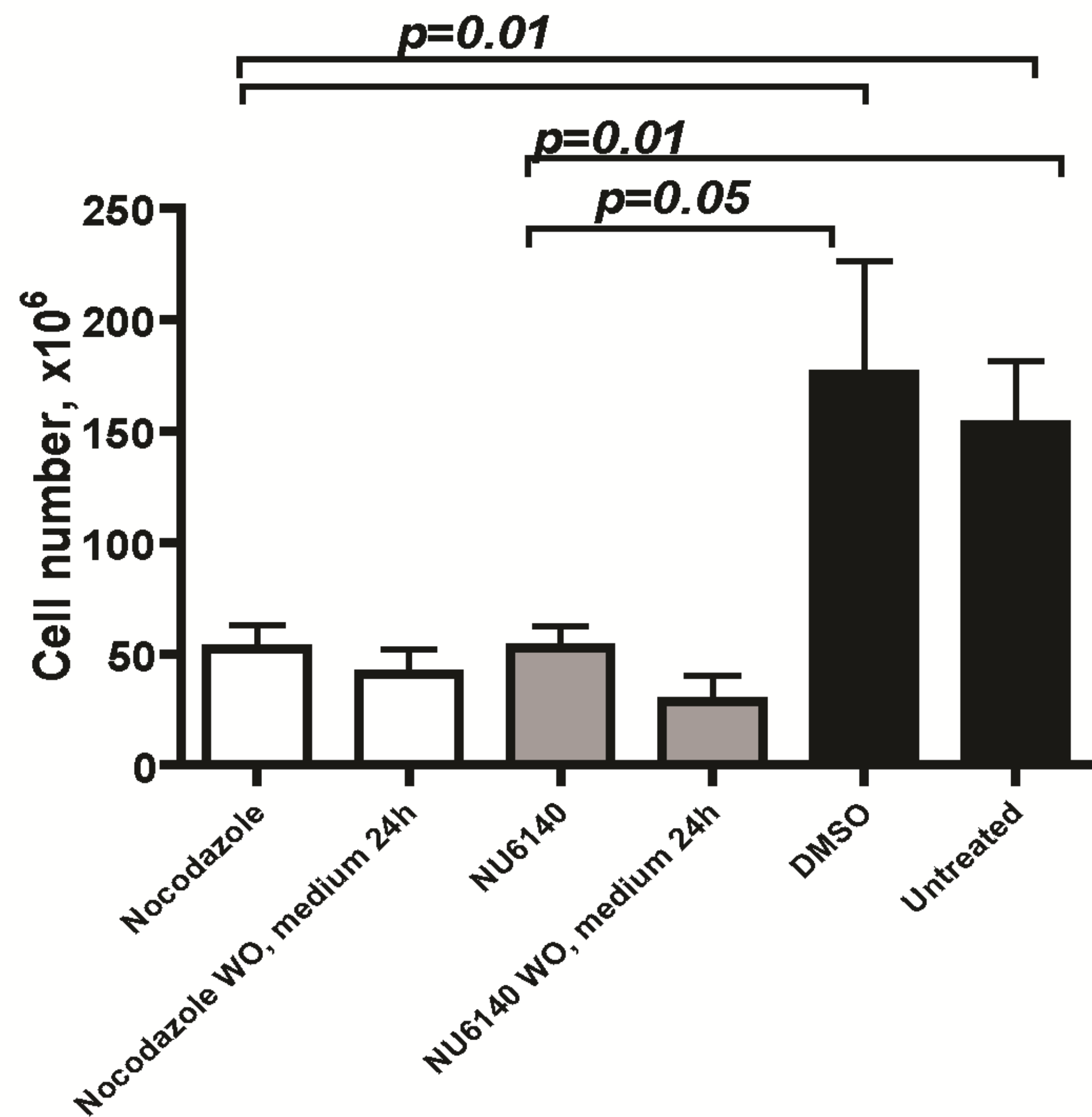**C**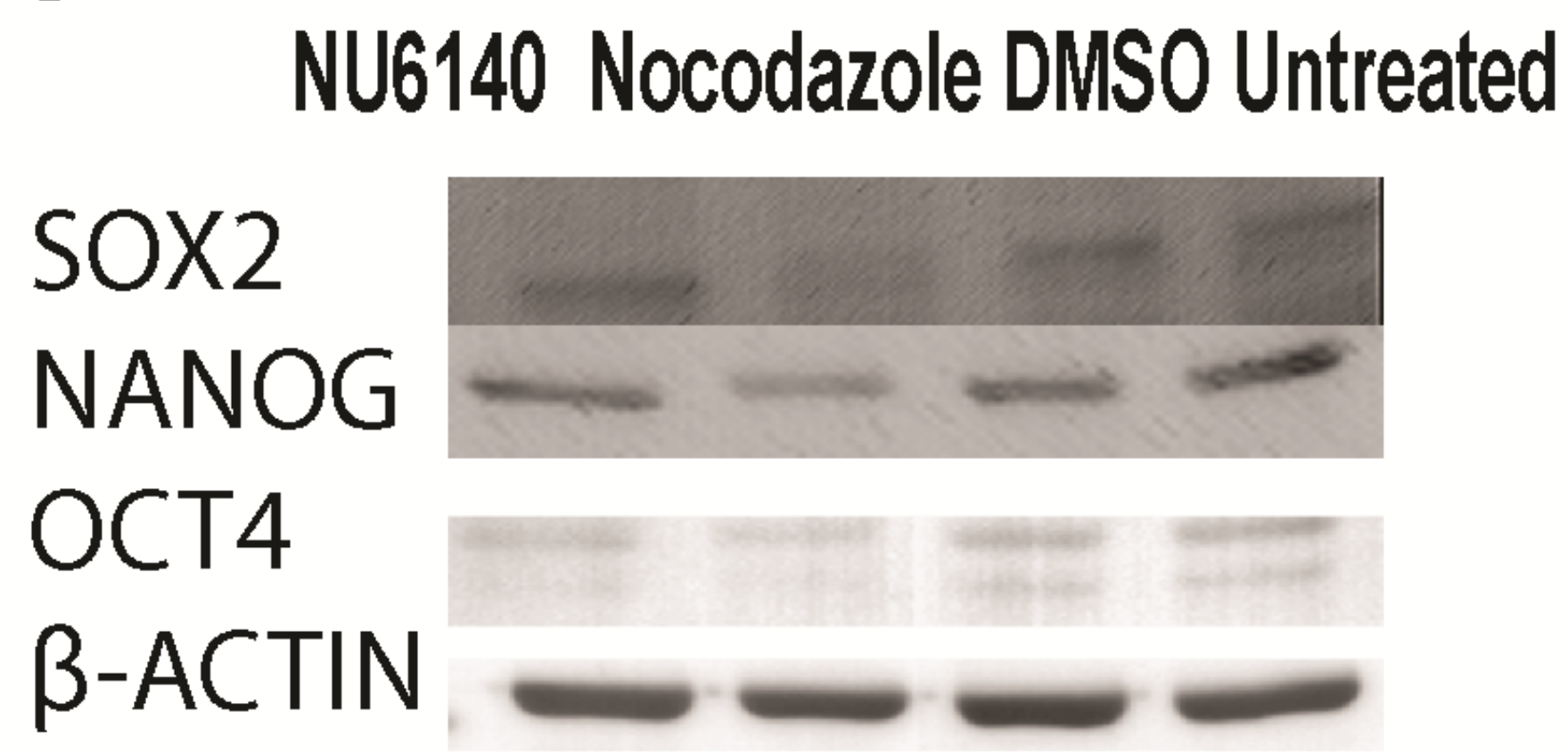**D**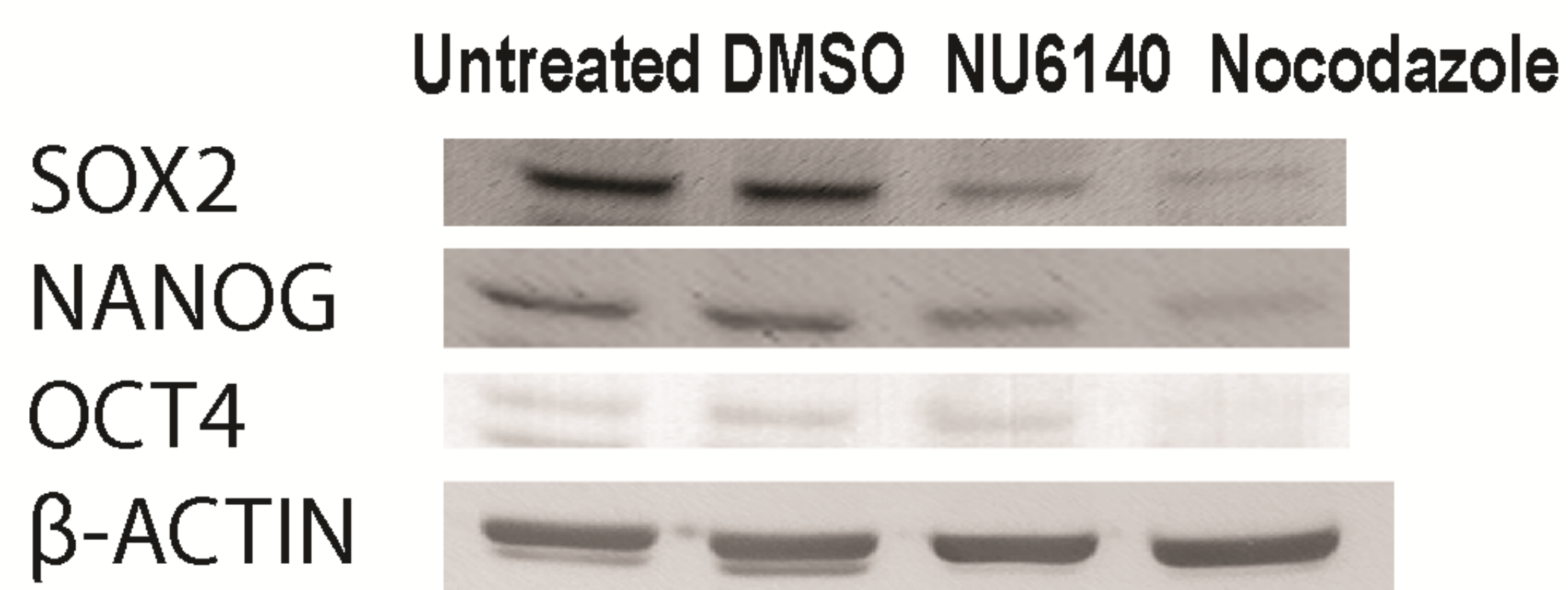

Supplement: Supplementary file 1 — Effects of NU6140 and nocodazole on hES and hEC cells survival and the expression of pluripotency markers NANOG, OCT4, SOX2 as detected by Western blotting method. [file 280638.f1.pdf]
